# Obituary

**Published:** 1847-12

**Authors:** 


### OBITUARY.

Dr. Wainwright of New York died recently from the bite of a rattle snake which had been sent to him as a curiosity, by a brother-in-law from Alabama.

Recent European news announces the death of Liston, the eminent English, and Diefenbach, the great German surgeons.

